# Predicting factors for abnormal brain computed tomography in children with minor head trauma

**DOI:** 10.1186/s12873-021-00540-1

**Published:** 2021-11-19

**Authors:** Taraneh Naghibi, Mina Rostami, Behrad Jamali, Zhaleh Karimimoghaddam, Alireza Zeraatchi, Asghar Jafari Rouhi

**Affiliations:** 1 Department of Anesthesiology and Critical Care Medicine, School of Medicine, Ayatollah Mousavi Hospital, Zanjan University of Medical Sciences, Zanjan, Iran; 2 Social Determinants of Health Research Center, Zanjan University of Medical Sciences, Zanjan, Iran; 3 Department of Emergency Medicine, School of Medicine, Valiasr-e-Asr Hospital, Ayatollah Mousavi Hospital, Zanjan University of Medical Sciences, Zanjan, Iran; 4 Department of Radiation Oncology, School of Medicine, Valiasr-e-Asr Hospital, Zanjan University of Medical Sciences, Zanjan, Iran

**Keywords:** Minor head trauma, Children, Traumatic brain injury, Brain computed tomography scan

## Abstract

**Background:**

Deciding whether a cranial Computed Tomography (CT) scan in a patient with minor head trauma (MHT) is necessary or not has always been challenging. Diagnosing Traumatic Brain Injury (TBI) is a fundamental part of MHT managing especially in children who are more vulnerable in terms of brain CT radiation consequences and TBI. Defining some indications to timely and efficiently predict the likelihood of TBI is necessary. Thus**,** we aimed to determine the impact of clinical findings to predict the need for brain CT in children with MHT.

**Methods:**

In a prospective cohort study, 200 children (2 to 14 years) with MHT were included from 2019 to 2020. The data of MHT-related clinical findings were gathered. The primary and secondary outcomes were defined as a positive brain CT and any TBI requiring neurosurgery intervention, respectively. In statistical analysis, we performed Binary Logistic regression analysis, Fisher’s exact test and independent samples t-test using SPSS V.26.

**Results:**

The mean age of participants was 6.5 ± 3.06 years. Ninety patients underwent brain CT. The most common clinical finding and injury mechanism were headache and falling from height, respectively. The results of brain CTs were positive in seven patients (3.5%). We identified three predicting factors for an abnormal brain CT including headache, decreased level of consciousness, and vomiting.

**Conclusion:**

We showed that repetitive vomiting (≥2), headache, and decreased level of consciousness are predicting factors for an abnormal brain CT in children with MHT.

## Key messages

1. The prevalence of Traumatic Brain Injury (TBI) in children with minor head trauma is considerably low.
2. Repetitive vomiting, headache, and decreased level of consciousness may be predictors of an abnormal brain CT in children with minor head trauma.
3. Falling from height is the most common mechanism of head trauma among children.

## Background

Head trauma, as one of the most common causes of emergency department (ED) visits, can have serious and fatal consequences including traumatic brain injury (TBI) [1]. Although not all types of head trauma lead to significant consequences, proper management and early detection of TBI is of paramount importance [2]. Annually, a significant number of TBI cases are diagnosed worldwide and notably a meaningful portion of which occur in low and middle-income countries (LMIC) countries [3]. Importantly, a substantial part of head trauma statistics is related to pediatric population which is estimated to have an incidence of 180–300 per 100,000 [4]. Among pediatric population, most cases of head trauma are minor (Glasgow Coma Scale (GCS) ≥14) and intracranial injuries are uncommon, so that on average 5% of minor head traumas (MHT) in children lead to TBI and of this less than 1% requires neurosurgical intervention [5]. Patients with MHT have a GSC of 14 to 15, and may initially experience transient loss of consciousness (LOC) or amnesia, but present without focal neurological defects on admission [4]. Cranial CT scan is the reference standard of diagnosing TBI. Deciding in which patients with MHT performing a brain CT is needed has always been challenging and appraisal of its necessity in the management of pediatric head trauma has been even more important [6].

Brain CT overuse imposes high costs on the healthcare system of countries especially in LMIC [3, 7]. Unfortunately, in such countries Access to CT is difficult and sometimes only available in capital cities. As a result, in such cases, patients are referred to big hospitals. Thus, requesting unnecessary brain CTs can lead to a remarkable burden on the patients and the healthcare systems. In addition, exposure to brain CT radiation might be associated with an increased susceptibility to leukemia and brain tumors in children [8].

There are signs and symptoms that increase likelihood of occurring TBI in children with head trauma (i.e. posttraumatic seizures, headache, amnesia, scalp hematoma, skull fracture, vomiting after head trauma and loss of consciousness) [1, 9]. Given that the most cases of head trauma are minor head trauma (89% of all cases) [1], and the fact that more than 90% of CT scans do not indicate brain injury [10], the need to use clinical findings to recognize which patients are more prone to TBI is crucial [11]. Moreover, it is important not to limit clinical judgment to head trauma decision-making rules alone, as their sensitivities and specificities have been shown to be low in some studies [12, 13]. Therefore, appraising the presence of TBI in children with MHT using the clinical findings acquired from history taking and physical examination is of great importance in order to avoid unnecessary brain CTs and predict TBIs more efficiently.

The objective of the current study was to identify the accuracy of clinical findings to predict the need for brain CT in children with MHT.

## Methods and materials

In a prospective study, we included 200 children with minor head trauma aged 2 to 14 years who had been referred to the ED of Ayatollah Mousavi Hospital in Zanjan, Iran from May 2019 to March 2020. The trauma ED of Ayatollah Mousavi Hospital is the reference hospital in the whole province which patients with head trauma and high suspicion of TBI are also referred to this ED from other cities of Zanjan that do not have brain CT facilities.

### Study subjects

Inclusion criteria were considered children aged 2 to 14 years, blunt head trauma within the first 24 h before the ED visit and GCS score of ≥14. We excluded all patients with a history of anticoagulant therapy, GCS ≤ 13, underlying cerebral diseases (i.e. brain tumors, ischemic or hemorrhagic lesions), penetrating head trauma, ventricular shunts and presenting to the ED after 24 h of head trauma.

Study design and protocol was approved by the Ethics Committee of Zanjan University of Medical Sciences [IR.ZUMS.REC.1398.024].

### Data collection and measurements

**Fig. 1:**
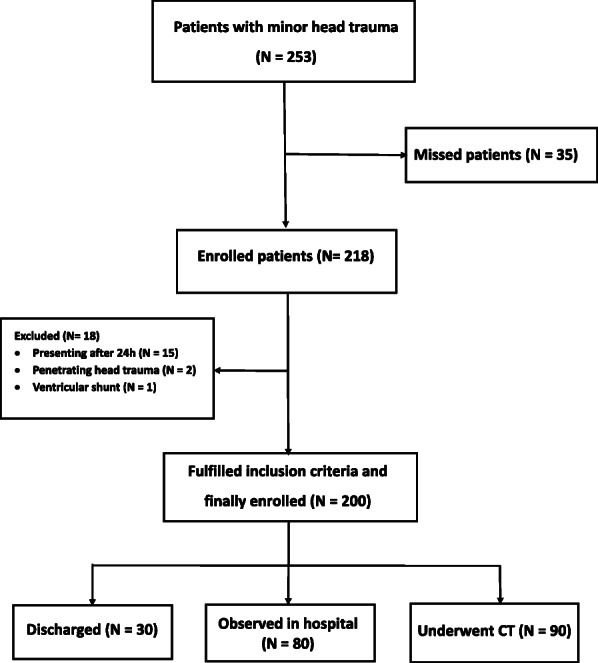
Study flow diagram

The data obtained from the history taking, physical examination and the results of brain CTs or follow ups were recorded in the forms designed for this purpose by emergency medicine physicians or residents. In fact, the study population was categorized into three groups (6 h follow up, brain CT, and discharge) according to ED physician’s decision on the basis of clinical signs and symptoms (Fig. 1). In other words, patients with high suspicion of TBI were referred to undergo a brain CT, patients with moderate suspicion of TBI were monitored in the ED for 6 h, and in case of any TBI-related signs and symptoms were referred to receive a brain CT. The rest of the patients who had very low clinical suspicion of TBI were discharged after explaining the TBI precautions (i.e. lowered level or loss of consciousness (LOC), persistent vomiting of at least two times, not acting normally to parents, cerebrospinal fluid rhinorrhea, and posttraumatic seizure) [1, 14] to their parents and they were asked to return promptly to the ED if any signs or symptoms relevant to TBI occurred. All CT images were interpreted by an experienced board certified radiologist.

Patients who did not undergo CT were followed up for one week after discharge and at the end of the seventh day were interviewed by an experienced nurse by phone calling to assess whether or not any TBI-related symptoms occurred, if there were any symptoms they were asked to undergo a cranial CT.

The scores of Pediatric Glasgow Coma Scale (PGCS) was used to assess the level of consciousness in children with MHT. PGCS evaluates children under 23 months, 2 to 5 years and over 5 years separately in three parts of verbal response, eye opening, and motor response [14].

Clinical findings of patients included repetitive vomiting (≥2), headache, decreased level of consciousness, posttraumatic seizure, posttraumatic amnesia, signs of skull base fracture (Battle sign, raccoon eyes, hemotympanum, and cerebrospinal fluid otorrhea or rhinorrhea), palpable skull fracture, scalp hematoma, acting abnormally to parents, and any bruising, abrasions or lacerations on face or scalp.

Additionally, we studied injury mechanisms included, fall from height, fall from stairs, ground level falls, hitting injury and any accident (pedestrian hit by car, bicycle-related accidents, motor vehicle accident, car rollover, and unrestrained).

### Outcome measures

Primary outcome was considered as an abnormal brain CT (i.e. pneumocephalus, cerebral contusion, subdural, epidural, ventricular or parenchymal hematoma, herniation, and skull fracture (depressed or linear)).

Secondary outcomes were any abnormality in brain CT requiring neurosurgery (i.e. elevation of skull fracture, craniotomy, intracranial pressure monitoring, and external ventricular drainage).

### Statistical analysis

In descriptive statistics, we reported continuous data as mean ± standard deviation (SD) or median with interquartile ranges (IQR), as applicable. Fisher’s exact test was used to evaluate the relationship between outcomes and predicting factors for categorical data. Independent samples t-test was used to compare the mean of continuous variables between two groups. We performed a binary Logistic regression analysis to determine which predicting factors can be considered as a risk indicator for a positive CT. Significance level was considered < 0.05. We performed all statistical analysis using SPSS V.26.

## Results

We included 200 children (134 males and 66 females) with MHT. The mean age of participants was 6.5 ± 3.06 years. Ninety patients received brain CT and none of the participants in the two other groups required a CT scan during the follow-up period.

Of participants, 76 (38%) showed no clinical findings. Headache (34%) was the most common clinical finding. Additionally, the most frequent injury mechanism was fall from height (37.5%) (Table 1).

**Table 1:** Basic characteristics of the participants

Variable	N (%) or mean ± SD
Gender, male	134 (67)
Age, year	6.5 ± 3.06
**Clinical findings**	
Repetitive vomiting	27 (13.5)
Headache	66 (33.0)
LOC	33 (16.5)
Posttraumatic amnesia	16 (8.0)
Scalp hematoma	17 (8.5)
Wound on face or scalp	33 (16.5)
Acting abnormally	6 (3.0)
Palpable skull fracture	0 (0)
Signs of skull base fracture	0 (0)
Posttraumatic seizure	0 (0)
**Trauma mechanism**	
Fall from height	75 (37.5)
Fall from stairs	34 (17.0)
Ground level fall	44 (22.0)
Bicycle-related accidents	16 (8.0)
Hitting injury	8 (4.0)
Motor vehicle accident	7 (3.5)
Car rollover	6 (3.0)
Unrestrained	10 (5.0)
Pedestrian hit by car	0 (0)

*LOC* Loss of Consciousness

We found 7 patients (3.5%) with positive brain CT including 1 patient with pneumocephalus, 2 with subdural hematoma, 1 with cerebral contusion, and 3 with linear skull fracture. However, none of the patients underwent neurosurgery (secondary outcome), 10 patients underwent neurosurgery counseling and were admitted to the neurosurgery ward to be monitored.

In examining the relationship between the clinical findings and abnormal brain CTs, there was a statistically significant relationship between headache, repetitive vomiting and decreased level of consciousness with abnormal brain CT (Table. 2).

**Table 2:** Relationship between clinical findings and abnormal brain CT

Variables	Abnormal CT N (%)		***P-***value^**a**^	LR
	Yes	No		
Male				
Yes	4 (3.0)	130 (97.0)	0.68	0.3
No	3 (4.5)	63 (95.5)		
Headache				
Yes	6 (9.1)	60 (90.9)	0.006	8.68
No	1 (0.7)	133 (99.3)		
Vomiting				
Yes	4 (14.8)	23 (85.2)	0.007	7.75
No	3 (1.7)	170 (98.3)		
LOC				
Yes	5 (15.2)	28 (84.8)	0.002	10.93
No	2 (1.2)	165 (98.8)		
Posttraumatic amnesia				
Yes	0 (0)	16 (100)	1.00	1.18
No	7 (3.8)	177 (96.2)		
Scalp hematoma				
Yes	0 (0)	17 (100%)	1.00	1.26
No	7 (3.8)	176 (96.2)		
Wound on face or scalp				
Yes	1 (3.0)	32 (97.0)	1.00	0.02
No	6 (3.6)	161 (96.4)		
Acting abnormally				
Yes	1 (16.7)	5 (83.3)	0.19	1.75
No	6 (3.1)	188 (96.9)		

^a^*P*-values obtained from Fisher’s exact test, *P* < 0.05

*LR* Likelihood Ratio, *CT* Computed Tomography, *LOC* Loss of Consciousness

In addition, no statistically significant relationship was found between injury mechanisms and abnormal brain CT (Table 3).

**Table 3:** Relationship between injury mechanisms and abnormal brain CT

Variables	Abnormal CT N (%)		***P-***value ^**a**^	LR
	Yes	No		
Fall from height				
Yes	4 (5.3)	71 (94.7)	0.42	1.14
No	3 (2.4)	122 (97.6)		
Fall from stairs				
Yes	0 (0)	34 (100)	0.6	2.66
No	7 (4.2)	159 (95.8)		
Ground level fall				
Yes	0 (0)	44 (100)	0.35	3.54
No	7 (4.5)	149 (95.5)		
Bicycle-related accidents				
Yes	0 (0)	16 (100)	1.00	1.18
No	7 (3.8)	177 (96.2)		
Hitting injury				
Yes	0 (0)	8 (100)	1.00	0.58
No	7 (3.6)	185 (96.4)		
Motor vehicle accident				
Yes	1 (14.3)	6 (85.7)	0.22	1.48
No	6 (3.1)	187 (96.9)		
Car rollover				
Yes	1 (16.7)	5 (83.3%)	0.19	1.75
No	6 (3.1)	188 (96.9%)		
Unrestrained				
Yes	1 (10.0)	9 (90.0)	0.3	0.91
No	6 (3.2)	184 (96.8)		

^a^*P*-values obtained from Fisher’s exact test, *P* < 0.05

*LR* Likelihood Ratio, *CT* Computed Tomography

In addition, no statistically significant relationship was found between injury mechanisms and abnormal brain CT (Table 3).

The results of independent samples t-test also revealed no significant relationship between age and an abnormal brain CT (t (198) = − 1.36, *P* = 0.17).

In order to determine the effect of independent variables to predict a positive cranial CT finding, we performed a binary logistic regression. Taking into account the fact that the number of predictors in our study were more than the number of events, we entered variables into the analysis using a forward selection method. The predicted variable was the presence or absence of a positive CT scan (7 events). Predictors were considered gender, age, repetitive vomiting, headache, LOC, posttraumatic amnesia, scalp hematoma, wound on face or scalp, acting abnormally, fall from height, fall from stairs, ground level fall, bicycle-related accidents, hitting injury, motor vehicle accident, Car rollover, unrestrained (Table 4).

**Table 4:** Predicting factors for an abnormal brain CT ^a^

Predictors ^**b**^	Coefficient	Standard error	OR (95% CI)	***P***-value
LOC	3.27	1.06	26.53 (3.28, 214. 34)	0.002
Vomiting	2.50	1.05	12.29 (1.57, 96.26)	0.017
Headache	2.49	1.18	12.10 (1.19, 122.47)	0.035
Constant	−6.95	1.44	0.001	0.000

^a^Predicting factors were defined using binary Logistic Regression with a forward selection method

^b^Only variables with a *p* < 0.05 were included in the model

*OR* Odds Ratio, *CI* Confidence Interval, *CT* Computed Tomography, *LOC* Loss of Consciousness

Finally, three predicting factors for abnormal brain CT including headache (*p* = 0.035), decreased level of consciousness (*p* = 0.002), and vomiting (*p* = 0.017) were recognized.

Indeed, these results imply that the presence of headache, vomiting and decreased level of consciousness in children with MHT might increase the odds of finding a positive cranial CT by more than 12 times, 12 times and 26 times, respectively.

For further analysis, we regarded the presence of clinical findings as well as the rate of different combinations of the three predicting factors we obtained (Table 5).

**Table 5:** Positive CT findings by number of three predicting factors

Predictors	Abnormal CT N (%)		Total N (%)	OR (95% CI)	***P***-value ^**a**^
**Number of clinical findings**	Yes	No			
**0**	0 (0)	76 (100)	76 (100)	0.60 (0.54, 0.67)	**0.046**
**≥1**	7 (5.6)	117 (94.4)	124 (100)		
**Presence of predictors**					
**Presence of predictors**					
**No**	0 (0)	100 (100)	100 (100)	1.07 (1.01, 1.13)	**0.014**
**Yes**	7 (7)	93 (93)	100 (100)		
**Presence of 1 predictor**					
**LOC**					
**Yes**	0 (0)	20 (100)	20 (100)	0.96 (0.93, 0.99)	1.000
**No**	7 (3.9)	173 (96.1)	180 (100)		
**Vomiting**					
**Yes**	0 (0)	13 (100)	13 (100)	0.96 (0.93, 0.99)	1.000
**No**	7 (3.7)	180 (96.3)	187 (100)		
**Headache**					
**Yes**	0 (0)	45 (100)	45 (100)	0.95 (0.92, 0.98)	0.353
**No**	7 (4.5)	148 (95.5)	155 (100)		
**Presence of 2 predictors**					
**Vomiting + Headache**					
**Yes**	2 (20.0)	8 (80.0)	10 (100)	9.25 (1.55, 55.18)	**0.041**
**No**	5 (2.6)	185 (97.4)	190 (100)		
**Vomiting + LOC**					
**Yes**	1 (100)	0 (0)	1 (100)	0.03 (0.01, 0.06)	**0.035**
**No**	6 (3)	193 (97)	199 (100)		
**Headache + LOC**					
**Yes**	3 (37.5)	5 (62.5)	8 (100)	28.20 (4.94, 160.75)	**0.001**
**No**	4 (2.1)	188 (97.9)	192 (100)		
**Presence of 3 predictors**					
**Vomiting + Headache + LOC**					
**Yes**	1 (33.3)	2 (66.7)	3 (100)	15.91 (1.26, 200.66)	0.102
**No**	6 (3.0)	191 (97.0)	197 (100)		

^a^*P*-values obtained from Fisher’s exact test, *P* < 0.05

*OR* Odds Ratio, *CI* Confidence Interval, *CT* Computed Tomography, *LOC* Loss of Consciousness

The results showed that 76 patients (37.8%) did not report any clinical findings. Besides, the presence of clinical findings was significantly associated with having an abnormal CT finding (*P* = 0.046), namely all 7 cases of abnormal cranial CT were reported in the group of patients with clinical findings.

In terms of patients reporting clinical findings (124 patients) there were 24 patients (11.9%) without 3 predicting factors. No patient with a positive CT was found among patients with none of the predictors so that presence of predicting factors was significantly associated with finding an abnormal CT in patients (*P* = 0.014).

Experiencing only one of the predictors (LOC or vomiting or headache) was not significantly associated with showing a positive CT (All, *p* > 0.05).

Considering the combinations of two predictors, all three showed a statistically significant association with having an abnormal CT (Table 5). According to the odds ratios, headache + LOC showed the greatest one equal to 28.20 (95% CI, 4.94–160.75), indicating that odds of finding an abnormal CT while the patient presents with headache + LOC seems to be nearly 28 times more than finding an abnormal CT when the patient does not present with headache + LOC. Similarly, in terms of vomiting + headache, with odds ratio equal to 9.25 (95% CI, 1.55–55.18), the odds of having an abnormal CT in patients with vomiting + headache might be approximately 9 times more than patients without this combination. Considering vomiting + LOC, the odds ratio less than 1, implies 0.03 odds of having an abnormal CT in patients with vomiting + LOC vs. patients without that combination. However, experiencing vomiting + headache + LOC was not statistically significantly associated with having an abnormal CT (*P* = 0.102), the odds ratio of 15.91 (95% CI, 1.26–200.66) signifies that the odds of finding an abnormal CT is nearly 15 times more in patients with this combination than those without it.

## Discussion

Minor head trauma is a common problem in children. In some cases, blunt MHT may be associated with TBI. Because the management and requesting brain CT in children with MHT is controversial, physicians usually tend to order a brain CT for most children with MHT, while TBI is seen only in a few cases. In the current study only 3.5% of children with MHT had positive brain CTs, similar to several other studies that have reported TBI in a very few cases with MHT [2, 4, 5].

Although the story is somewhat different in developing countries, Norlund et al showed that CT was a more cost-effective strategy than monitoring MHT patients at ED [15]. In addition, Geijerstam et al reported that CT was an attainable strategy in MHT patients and resulted in similar outcomes in comparison with observation [16]. Nevertheless, to assess the necessity of ordering brain CT in children with MHT is not just about the costs to healthcare system or the availability of CT, it is also important not to expose children to radiation as it can increase the risk of malignancy in them. It should be noted that the use of CT in children is more than adults and is on the rise. This can have various reasons, such as the physician’s fear of missing a TBI in children with MHT or the difficulties of monitoring children at ED [17, 18].

We observed a statistically significant association between repetitive vomiting, decreased level of consciousness, and headache with abnormal brain CT whereas there was not any significant relationship between mechanisms of injury and a positive brain CT. It implies that repetitive vomiting, decreased consciousness and headache might be three predicting factors for an abnormal brain CT in children presenting with MHT.

Emerging evidence has confirmed the importance of vomiting, especially repetitive vomiting (2 ≤), as a risk indicator for TBI [1]. Vaniyapong et al in their study have demonstrated repetitive vomiting and headache as clinical predictors for TBI [19].

However, in many pediatric head trauma guidelines repetitive vomiting has been indicated as a predicting factor for TBI, there is ample evidence that no association exists between TBI and vomiting. Interestingly, it has been concluded that vomiting in children after head trauma may be more closely related to individual or familial predisposition than to the presence of TBI [20]. Moreover, Studies have shown that there is a significant difference between isolated vomiting and non-isolated vomiting as a factor in predicting TBI likelihood, so that isolated vomiting is not significantly associated with TBI [21]. Similar to the present study that none of the 7 patients with abnormal CT had isolated symptoms.

Headache and decreased level of consciousness are also clinical findings that may help predict TBI in patients with MHT. Nonetheless, this is also controversial and some evidence has revealed conflicting results in terms of headache and decreased consciousness as risk indicators for MHT [1]. Sharif-Alhoseini et al in a study of risk indicators for abnormal brain CT have determined headache and loss of consciousness as clinical findings to predict a positive CT in patients with MHT [22]. In addition, there are other studies that have shown headache, loss of consciousness, and vomiting moderately increase the probability of an abnormal CT in children with MHT [23].

Another finding of the present study is the highest frequency of falling from height among the mechanisms of trauma. It has been demonstrated that in the age group of 0–14 years, the main mechanism of trauma is falling from height [14].

Although according to our findings there was no significant relationship between gender and an abnormal CT, various studies indicate that there is a significant difference in the likelihood of TBI between boys and girls, so that boys are about 2 times more likely to develop TBI after a head trauma than girls [14, 24].

### Limitations

The main limitation of the present study is the low sample size and therefore the low number of positive CT findings that can limit the power of the current analysis. However, over a one-year period, the authors included all eligible minor head trauma cases who referred to the only trauma center of the Zanjan province.

## Conclusion

In summary, we observed abnormal brain CT in only 3.5% of children with MHT. Repetitive vomiting, decreased level of consciousness, and headache were clinical findings that we showed to be significantly associated with a positive CT. Thus, we found 3 predicting factors for abnormal brain CT in children with MHT.

## Data Availability

The datasets used and analyzed during the current study are available from the corresponding author on reasonable request.

## Abbreviations

CT: Computed Tomography
MHT: Minor Head Trauma
TBI: Traumatic Brain Injury
ED: Emergency Department
LMIC: Low and Middle-Income Countries
GCS: Glasgow Coma Scale
LOC: Loss Of Consciousness
PGCS: Pediatric Glasgow Coma Scale
SD: Standard Deviation
IQR: Inter Quartile Range
LR: Likelihood Ratio
